# Comparative analysis of two complete *Corynebacterium ulcerans *genomes and detection of candidate virulence factors

**DOI:** 10.1186/1471-2164-12-383

**Published:** 2011-07-30

**Authors:** Eva Trost, Arwa Al-Dilaimi, Panagiotis Papavasiliou, Jessica Schneider, Prisca Viehoever, Andreas Burkovski, Siomar C Soares, Sintia S Almeida, Fernanda A Dorella, Anderson Miyoshi, Vasco Azevedo, Maria P Schneider, Artur Silva, Cíntia S Santos, Louisy S Santos, Priscila Sabbadini, Alexandre A Dias, Raphael Hirata, Ana L Mattos-Guaraldi, Andreas Tauch

**Affiliations:** 1 Institut für Genomforschung und Systembiologie, Centrum für Biotechnologie, Universität Bielefeld, Universitätsstraße 27, D-33615 Bielefeld, Germany; 2 CLIB Graduate Cluster Industrial Biotechnology, Centrum für Biotechnologie, Universität Bielefeld, Universitätsstraße 27, D-33615 Bielefeld, Germany; 3 Bioinformatics Resource Facility, Centrum für Biotechnologie, Universität Bielefeld, Universitätsstraße 25, D-33615 Bielefeld, Germany; 4 Lehrstuhl für Genomforschung, Fakultät für Biologie, Universität Bielefeld, Universitätsstraße 27, D-33615 Bielefeld Germany; 5 Lehrstuhl für Mikrobiologie, Friedrich-Alexander-Universität Erlangen-Nürnberg, Staudtstraße 5, D-91058 Erlangen, Germany; 6 Laboratório de Genética Celular e Molecular, Departamento de Biologia Geral, Instituto de Ciências Biológicas, Universidade Federal de Minas Gerais, Av. Antonio Carlos 6627, Pampulha, Belo Horizonte, MG, Brazil; 7 Instituto de Ciências Biológicas, Universidade Federal do Pará, Rua Augusto Corrêa, 01-Guamá, Belém, PA, Brazil; 8 Faculdade de Ciências Médicas, Universidade do Estado do Rio de Janeiro, Av. 28 de Setembro 87, 20551-030 Rio de Janeiro, RJ, Brazil

## Abstract

**Background:**

*Corynebacterium ulcerans *has been detected as a commensal in domestic and wild animals that may serve as reservoirs for zoonotic infections. During the last decade, the frequency and severity of human infections associated with *C. ulcerans *appear to be increasing in various countries. As the knowledge of genes contributing to the virulence of this bacterium was very limited, the complete genome sequences of two *C. ulcerans *strains detected in the metropolitan area of Rio de Janeiro were determined and characterized by comparative genomics: *C. ulcerans *809 was initially isolated from an elderly woman with fatal pulmonary infection and *C. ulcerans *BR-AD22 was recovered from a nasal sample of an asymptomatic dog.

**Results:**

The circular chromosome of *C. ulcerans *809 has a total size of 2,502,095 bp and encodes 2,182 predicted proteins, whereas the genome of *C. ulcerans *BR-AD22 is 104,279 bp larger and comprises 2,338 protein-coding regions. The minor difference in size of the two genomes is mainly caused by additional prophage-like elements in the *C. ulcerans *BR-AD22 chromosome. Both genomes show a highly similar order of orthologous coding regions; and both strains share a common set of 2,076 genes, demonstrating their very close relationship. A screening for prominent virulence factors revealed the presence of phospholipase D (Pld), neuraminidase H (NanH), endoglycosidase E (EndoE), and subunits of adhesive pili of the SpaDEF type that are encoded in both *C. ulcerans *genomes. The *rbp *gene coding for a putative ribosome-binding protein with striking structural similarity to Shiga-like toxins was additionally detected in the genome of the human isolate *C. ulcerans *809.

**Conclusions:**

The molecular data deduced from the complete genome sequences provides considerable knowledge of virulence factors in *C. ulcerans *that is increasingly recognized as an emerging pathogen. This bacterium is apparently equipped with a broad and varying set of virulence factors, including a novel type of a ribosome-binding protein. Whether the respective protein contributes to the severity of human infections (and a fatal outcome) remains to be elucidated by genetic experiments with defined bacterial mutants and host model systems.

## Background

Toxigenic *Corynebacterium ulcerans *was first isolated from a throat lesion of a patient with respiratory diphtheria-like illness in 1926 [1]. These *C. ulcerans *strains produce a diphtheria toxin, which is similar to that encoded by toxigenic strains of *Corynebacterium diphtheriae *[2,3]. This observation has been explained by the fact that *C. ulcerans *may harbor lysogenic β-corynephages coding for the diphtheria toxin, which is responsible for the systemic symptoms caused by *C. diphtheriae *[4]. Respiratory diphtheria-like illnesses caused by toxigenic *C. ulcerans *strains are increasingly reported from various industrialized countries [5] and became more common than *C. diphtheriae *infections in the United Kingdom [6]. Human infections with toxigenic *C. ulcerans *can be fatal in unvaccinated patients and usually occur in adults, who consumed raw milk [7,8] or had close contact with domestic animals [6]. *C. ulcerans *has been detected as a commensal not only in domestic animals, but also in wild animals, implying that both groups may serve as reservoirs for the zoonotic transmission of this pathogen [9,10]. Several reports demonstrated that toxigenic *C. ulcerans *strains can be recovered from dairy cows, cats, dogs, goats, pigs, squirrels, free-living otters, dromedary camels, and macaques [5,11]. Moreover, *C. ulcerans *isolates from domestic cats were found to exhibit the predominant ribotypes observed among human clinical isolates, suggesting that strains isolated from cats are a potential reservoir for human infection [12]. Likewise, ribotyping of *C. ulcerans *from a female diphtheria patient and a chronic labial ulcer of her dog revealed that both isolates correspond to a single strain [9]. This example demonstrates that a distinct *C. ulcerans *strain may infect different hosts.

Beside respiratory diphtheria-like illnesses, *C. ulcerans *can also cause extrapharyngeal infections in humans, including severe pulmonary infections [13-15]. When *C. ulcerans *isolates from human clinical specimens not fitting reporting criteria for cases of diphtheria were tested for the presence of diphtheria toxin only a portion of strains were positive for the *tox *gene encoding diphtheria toxin [16,17]. These observations indicate that additional factors contribute to the virulence of "non-toxigenic" *C. ulcerans *strains. A second dermonecrotic toxin with similarity to toxic phospholipase D (Pld) from *Corynebacterium pseudotuberculosis *appeared to be characteristic of *C. ulcerans *[18]. The common repertoire of potent toxins in *C. diphtheriae*, *C. ulcerans *and *C. pseudotuberculosis *particularly highlights the close phylogenetic relationship between these three species [19]. Despite this apparent relationship, levels of genomic DNA relatedness and taxonomic analyses of 16S rDNA sequences showed that *C. diphtheriae*, *C. ulcerans *and *C. pseudotuberculosis *are separate taxa within a distinct cluster of the genus *Corynebacterium *[19]. They can be separated clearly from other pathogenic corynebacteria by chemotaxonomic assays [20]. Hart *et al*. proposed that the three species evolved from a common ancestor, which parasitized in ungulates in pre-human times [8].

Although *C. ulcerans *and *C. pseudotuberculosis *are of increasing medical importance, very little knowledge of their lifestyles and associated virulence factors was available until recently. We extended the genetic knowledge of this corynebacterial cluster by publishing the annotation of four complete genome sequences from *C. pseudotuberculosis *strains isolated from goat, sheep, cattle, and a rare case of human lymphadenitis [21-23]. In conjunction with the previously evaluated genome sequence from the toxigenic *C. diphtheriae *strain NCTC 13129 [24], a more detailed picture of the closely related corynebacterial pathogens is now available at the genetic and genomic level. In the present study, we established the genome sequences of two *C. ulcerans *strains (809 and BR-AD22) from human and animal specimens to characterize the architecture of the genome and to compare the predicted gene contents and the repertoires of candidate virulence factors.

*C. ulcerans *809 was recovered from a bronchoalveolar lavage (BAL) sample of an 80-year-old woman with rapidly fatal pulmonary infection and a history of chronic bilateral limb ulcers [15]. The woman lived in the metropolitan area of Rio de Janeiro and was hospitalized in coma, with shock and acute respiratory failure. Cultures from the BAL sample revealed the presence of *C. ulcerans*, and varying antimicrobial therapies were directed to this potential pathogen. This medical treatment resulted in complete healing of the skin lesions. Nevertheless, the cardiorespiratory symptoms of the patient worsened and medical examinations showed evidence of multiple organ failures. The patient died 23 days after hospitalization. Subsequent toxigenicity tests and PCR assays evaluating the production of diphtheria toxin by *C. ulcerans *809 were ambiguous and it was concluded that the unusual nature of the pathogen possibly contributed to the patient's death [15].

The second isolate investigated in this study, *C. ulcerans *BR-AD22, was obtained from a nasal sample of a 5-year-old female dog kept in an animal shelter in the metropolitan area of Rio de Janeiro [25]. General clinical aspects and laboratory findings revealed this dog as an asymptomatic carrier of *C. ulcerans*. Toxigenicity assays showed the presence of phospholipase D in *C. ulcerans *BR-AD22, but were negative for the presence of diphtheria toxin [25]. In the following sections, we present the results of the genome sequencing project and the comparative analysis of the genomes from the selected *C. ulcerans *strains, thereby focusing on the relevant differences in the gene content and the repertoires of virulence factors.

## Results and discussion

### General features and architecture of the *C. ulcerans *genomes

**Figure 1:**
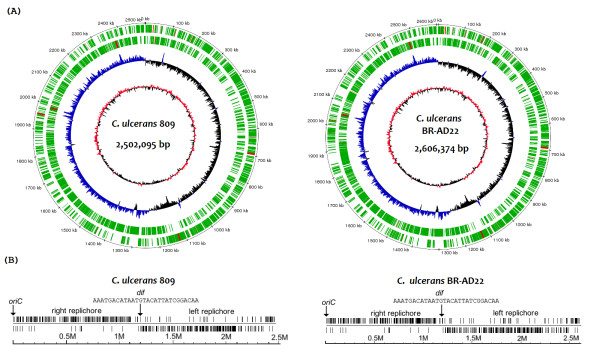
**The complete genomes of *C. ulcerans *809 and *C. ulcerans *BR-AD22**. **(A)**, Circular representation of the chromosomes from *C. ulcerans *809 and *C. ulcerans *BR-AD22. The circles represent the following features: circle 1, DNA base position; circles 2 and 3, predicted coding sequences transcribed clockwise and anticlockwise, respectively; circle 4, G/C skew [(G-C)/(G+C)] plotted using a 10-kb window; circle 5, G+C content plotted using a 10-kb window. Color code in circles 2 and 3: green, predicted protein-coding regions; red, rRNA or tRNA genes. **(B)**, Distribution of actinobacterial architecture imparting sequences on the leading and lagging strands of the two *C. ulcerans *chromosomes. The deduced position of the putative *dif *region is indicated in the linear representation of the chromosomes. The position of the origin of replication (*oriC*) and the nucleotide sequence of the conserved 28-bp sequence of the *dif *region are indicated.

The genome sequences of *C. ulcerans *809 from a human clinical source and *C. ulcerans *BR-AD22 from an asymptomatic dog were determined by pyrosequencing using a quarter of a sequencing run with the Genome Sequencer FLX Instrument for each strain. The resulting reads were assembled with the Newbler Assembler software, and the remaining gaps were closed by PCR strategies that were supported by the related reference contig arrangement tool r2cat [26] using the genome sequence of *C. pseudotuberculosis *FRC41 as a reference [21]. The final assemblies of the two genomic DNA sequences yielded circular chromosomes with a mean G+C content of 53% (Figure 1A), which is very similar to that of the closely related species *C. pseudotuberculosis *(52.2%) [21] and *C. diphtheriae *(53.5%) [24]. The chromosome of *C. ulcerans *809 has a size of 2,502,095 bp and is thus 104,279 bp smaller than that of *C. ulcerans *BR-AD22 (Table 1). The subsequent gene prediction and annotation of the genome sequences was performed automatically with the GenDB system [27]. After manual curation of the annotation, 2,182 protein-coding regions were detected in the *C. ulcerans *809 genome, whereas 2,338 protein-coding regions were predicted in the genome sequence of *C. ulcerans *BR-AD22. These data already indicated strain-specific differences in the gene repertoires of both *C. ulcerans *isolates. Relevant data and general features deduced from the genome sequences of *C. ulcerans *809 and *C. ulcerans *BR-AD22 are summarized in Table 1.

**Table 1:** General features of the genome sequences of *C. ulcerans *809 and *C. ulcerans *BR-AD22

Feature	*C. ulcerans *809	*C. ulcerans *BR-AD22
Genome size (bp)	2,502,095	2,606,374
Sequenced bases	106,993,163	59,757,327
Genome coverage	42.8 ×	22.9 ×
G+C content (%)	53.3	53.4
Coding sequences	2,182	2,338
Coding density (%)	87.7	87.8
Average gene length (bp)	1,006	979
ribosomal RNAs	4 × (16S-23S-5S)	4 × (16S-23S-5S)
transfer RNAs	52	52
Prophages	1	4
CRISPRs^a^	3 loci	3 loci

^a ^Abbreviation: Clustered Regularly Interspaced Short Palindromic Repeats

The calculation of the G/C skew [(G-C)/(G+C)] of both genome sequences revealed a bi-directional replication mechanism for the *C. ulcerans *chromosome (Figure 1A). According to the presence of conserved DnaA boxes [TTATC(C/A)A(C/A)A], the origin of chromosomal replication (*oriC*) is located downstream of the *dnaA *gene and has a computed length of 423 bp [28]. The plot of the G/C skew additionally indicated the presence of a putative *dif *region involved in replication termination [29] at the expected position of about 180° from *oriC*, dividing the chromosome of *C. ulcerans *in two replichores of similar size (Figure 1A). For a more precise detection of the *dif *region, the distribution of the actinobacterial architecture imparting sequences G(A/T/C)GGGGGA and (T/C)GGGGGAG [30] was plotted on the leading and lagging strands of the *C. ulcerans *chromosomes (Figure 1B). Both linear plots show a characteristic distribution of the architecture imparting sequences, as these octamers are overrepresented on the leading strands and underrepresented on the lagging strands. Putative *dif *regions were detected at around 1,193 kb of the chromosomal map of *C. ulcerans *809 and at 1,195 kb of the *C. ulcerans *BR-AD22 chromosome (Figure 1B). In accordance with this computation, the respective DNA regions of the *C. ulcerans *chromosomes contain a conserved 28-bp sequence that shows similarity to the consensus sequence of actinobacterial *dif *sites [29]. Moreover, four *rrn *operons were identified in the genome sequences of *C. ulcerans *809 and *C. ulcerans *BR-AD22. All *rrn *operons are located on the leading strands of the *C. ulcerans *chromosome; two are present on the right and two on the left replichore, respectively. In summary, the structural analysis of the complete genome sequences of two *C. ulcerans *strains revealed the typical architecture of a corynebacterial chromosome in this species [31,32] with the presence of strain-specific variations that were investigated in more detail by the following comparative analysis.

### Comparative analysis of the gene order in the *C. ulcerans *genomes

**Figure 2:**
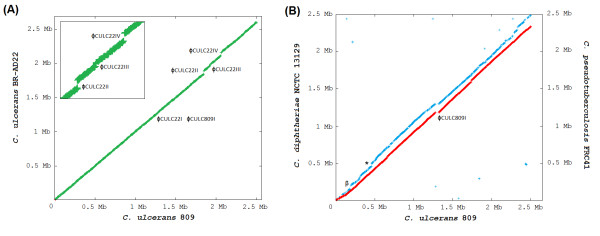
**Comparative analysis of the gene order in *C. ulcerans*, *C. diphtheriae *and *C. pseudotuberculosis***. **(A)**, Synteny between the sequenced chromosomes of *C. ulcerans *809 and *C. ulcerans *BR-AD22. **(B)**, Synteny between the chromosome of *C. ulcerans *809 and those from *C. diphtheriae *NCTC 13129 (blue) and *C. pseudotuberculosis *FRC41 (red). The graphs represent X-Y plots of dots forming syntenic regions between the selected chromosomes. Each dot represents a predicted protein having an orthologous counterpart in another corynebacterial genome, with co-ordinates corresponding to the position of the respective coding region in each genome. Orthologous proteins were detected by reciprocal best BLASTP matches. The genomic positions of putative prophages detected in *C. ulcerans *are marked in the synteny plots. Symbols: β, β-corynephage of *C. diphtheriae *NCTC 13129; asterisk, nitrate reductase gene region of *C. diphtheriae *NCTC 13129.

A synteny analysis was performed by plotting reciprocal best BLASTP matches [33] to compare the gene order in the chromosomes of *C. ulcerans *809 and *C. ulcerans *BR-AD22. This computation revealed a highly conserved order of orthologous genes between the two *C. ulcerans *chromosomes, as only three breakpoints of synteny were detectable in the left replichore (Figure 2A). These breakpoints are clearly indicative of the insertion of additional DNA regions into the chromosome of *C. ulcerans *BR-AD22. The annotation of the respective gene regions suggests that the breakpoints are due to the presence of prophage-like elements in the *C. ulcerans *BR-AD22 genome, named ΦCULC22II, ΦCULC22III, and ΦCULC22IV (Figure 2A). An additional prophage-like region (ΦCULC809I and ΦCULC22I) is present at identical positions in both *C. ulcerans *chromosomes (Figure 2A). Considering a total size of 99.9 kb for the three additional prophage-like elements in *C. ulcerans *BR-AD22 (Table 2), the difference in size of the two *C. ulcerans *genomes is mainly caused by variations in the individual repertoire of prophages. This result indicates that the sequenced *C. ulcerans *strains are very closely related, although they were originally isolated at different locations from a human clinical specimen and the nares of an asymptomatic dog.

**Table 2:** General features of prophage-like elements detected in the *C. ulcerans *genomes

Name	Size	G+C content	No. of CDS	CDS with assigned function	Integration site	Attachment site
ΦCULC809I	41.4 kb	53%	45	17	CULC809_01141	Not detected
ΦCULC22I	42 kb	53%	42	13	CULC22_01157	Not detected
ΦCULC22II	44.9 kb	55%	60	18	Between CULC22_01663 and CULC22_01724	TTAGATAC
ΦCULC22III	14 kb	57%	19	9	tRNA^Lys^	TTCAAGTCCCTGATGGCGCAC
ΦCULC22IV	41 kb	54%	53	16	tRNA^Thr^	TTGAGCTGGAGATGGGACTTGAACCC

The gene order in *C. ulcerans *809 was moreover compared to those in the taxonomically closely related species *C. diphtheriae *and *C. pseudotuberculosis*. This comparison revealed also a highly conserved order of orthologous genes in the genomes of *C. diphtheriae *NCTC 13129 and *C. pseudotuberculosis *FRC41 (Figure 2B), which is consistent with the previous observation that genetic rearrangements are rare in genomes of species belonging to the main lineage of the genus *Corynebacterium *[32]. Only one remarkable breakpoint of synteny was observed when comparing the order of genes in *C. ulcerans *809 and *C. pseudotuberculosis *FRC41. This breakpoint is located at 1,300 kb of the chromosomal map of *C. ulcerans *809 and is caused by the integration of the prophage-like element ΦCULC809I (Figure 2B).

The synteny analysis between *C. ulcerans *809 and *C. diphtheriae *NCTC 13129 revealed two additional breakpoints within the highly conserved order of genes. The first breakpoint is located about 154 kb downstream of *oriC *and comprises the genes of the corynephage β (DIP0180 to DIP0222), among others coding for the diphtheria toxin in *C. diphtheriae *NCTC 13129. The lysogenic β-corynephage is completely missing in the genome of *C. ulcerans *809. The second breakpoint of synteny is located at about 457 kb and comprises genes for nitrate reductase and associated protein-coding regions for the synthesis of the molybdenum cofactor (DIP0492 to DIP0507) [24]. This observation is in line with taxonomic reports using nitrate reductase activity as a distinct metabolic marker to distinguish between *C. diphtheriae *(*gravis*, *intermedius*, *mitis*) and *C. ulcerans *isolates, as nitrate reductase activity was not detectable in the latter species [20].

### Comparative analysis of prophage-like sequences in the *C. ulcerans *genomes

**Figure 3:**
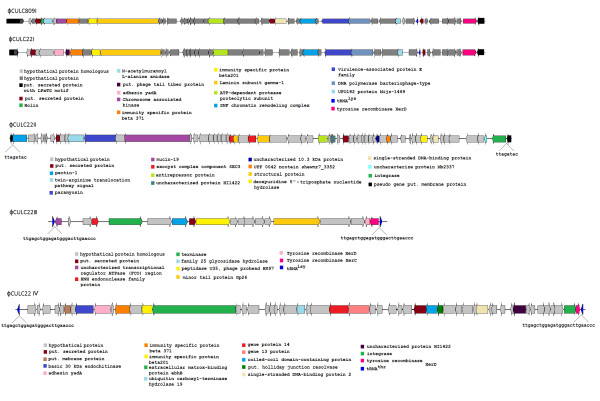
**Genetic maps of putative prophages detected in the *C. ulcerans *genomes**. The functional annotation of the prophage-like region ΦCULC809I from *C. ulcerans *809 and of the prophage-like regions ΦCULC22I, ΦCULC22II, ΦCULC22III, and ΦCULC22IV from *C. ulcerans *BR-AD22 is shown. The predicted protein functions are indicated by color codes. ΦCULC809I and ΦCULC22I are closely related genetic elements according to the very high overall similarity of their genes and gene products. The nucleotide sequences of putative integration sites of ΦCULC22II, ΦCULC22III, and ΦCULC22IV in the chromosome of *C. ulcerans *BR-AD22 are shown. tRNA genes flanking the putative prophages are indicated as blue triangles.

According to the synteny analysis, the variation in the repertoire of prophage-like regions is a remarkable difference between the sequenced *C. ulcerans *genomes, as one putative prophage was identified in the *C. ulcerans *809 genome and four prophage-like regions were detected in the genome sequence of *C. ulcerans *BR-AD22 (Table 2). Accordingly, this genome project provides the first molecular genetic data about corynephages infecting the species *C. ulcerans *that may also harbor β-corynephages coding for the diphtheria toxin [34]. The respective genomic regions of *C. ulcerans *809 and *C. ulcerans *BR-AD22 were characterized in more detail and the deduced genetic maps of the putative prophages are presented in Figure 3.

The prophage-like regions ΦCULC22I from *C. ulcerans *BR-AD22 and ΦCULC809I from *C. ulcerans *809 have a size of about 42 kb and are characterized by highly similar genetic maps. Both prophage-like elements were detected at the same genomic position and apparently integrated at slightly different sites into a coding region for a hypothetical protein (CULC22_01157 and CULC809_01141) that may represent the integration site of these phages in the *C. ulcerans *chromosome. Minor differences between the putative prophages were detected in the number of predicted genes: the ΦCULC22I region comprises 42 genes (CULC22_01158 to CULC22_01199), whereas 45 genes were assigned to the ΦCULC809I region (CULC809_01142 to CULC809_01186). According to BLASTP matches and global amino acid sequence alignments, both putative prophages share 36 genes that code for gene products with a least 98% amino acid sequence identity. These values clearly demonstrate the very close relationship of both prophages from different *C. ulcerans *strains.

The prophage-like region ΦCULC22II from *C. ulcerans *BR-AD22 has a size of 44.9 kb and comprises 60 genes (Figure 3). A sequence comparison with the corresponding region in the genome of *C. ulcerans *809 revealed that this putative prophage is apparently integrated into the ortholog of CULC809_01647 encoding a hypothetical protein. The integration of the putative phage into the chromosome of *C. ulcerans *BR-AD22 probably divided this gene into two pseudogenes (CULC22_01663 and CULC22_01724) that are located directly adjacent to the ΦCULC22II region. This view is supported by the presence of 8-bp direct repeats that are located at the borders of this prophage-like element and may represent the integration site of ΦCULC22II in *C. ulcerans *(Figure 3).

The third prophage-like region of *C. ulcerans *BR-AD22 (ΦCULC22III) is located adjacent to a second tRNA^Lys ^gene that may represent the integration site due to the presence of a 21-bp direct repeat, which is part of this tRNA^Lys ^gene and flanks the prophage-like region (Figure 3). The ΦCULC22III region has a size of about 14 kb and comprises 19 genes (CULC22_01793 to CULC22_01811). The size difference between the prophage-like regions of *C. ulcerans *BR-AD22 suggests that at least ΦCULC22III is incomplete and a defective remnant of a formerly active corynephage (Table 2). The prophage-like region ΦCULC22IV of *C. ulcerans *BR-AD22 comprises 53 genes (CULC22_01925 to CULC22_01977), has a size of about 41 kb and is located adjacent to a tRNA^Thr ^gene. A 26-bp direct repeat, which is part of the tRNA gene, may represent the integration site of the respective corynephage.

The previous experimental characterization of *C. ulcerans *809 suggested the presence of a β-corynephage in this strain, as PCR assays amplified putative fragments of the *tox *gene coding for the diphtheria toxin [15]. However, neither the *tox *gene nor other DNA segments of the β-corynephage were identified in the genome sequences of *C. ulcerans *809 and *C. ulcerans *BR-AD22. This result is also obvious when considering the lack of synteny between the chromosomes of *C. diphtheriae *NCTC 13129 and *C. ulcerans *809 at the integration site of the corynephage β (Figure 2B). Nevertheless, remnants of a putative corynephage are located adjacent to the tRNA^Arg ^gene, which comprises the attachment site for the β-phage [35]. The genes CULC809_00176 and CULC22_00173 code for putative phage-type integrases in the genome sequence of *C. ulcerans *BR-AD22 and *C. ulcerans *809. The respective tyrosine recombinase from *C. ulcerans *shares 92% amino acid sequence identity with the integrase of the β-corynephage integrated in the genome of *C. diphtheriae *NCTC 13129 and is encoded adjacent to the tRNA^Arg ^gene. This gene annotation supports the assumption that a lysogenic β-corynephage-like phage had been integrated in both *C. ulcerans *genomes in former times.

### Detection and comparative analysis of CRISPR regions in the *C. ulcerans *genomes

A screening of the genome sequences of *C. ulcerans *809 and *C. ulcerans *BR-AD22 with the CRISPRFinder program [36] revealed the presence of three loci of so-called clustered regularly interspaced short palindromic repeats (CRISPRs) (Table 3). These repeat regions are often associated with CRISPR-associated (*cas*) genes and may provide acquired immunity against bacteriophages and other foreign genetic elements by means of a sequence specificity that is determined by similarities between the spacer sequences and foreign DNA [37]. CRISPR locus I is present in both sequenced *C. ulcerans *genomes and flanked by four *cas *genes (CULC809_00031 to CULC809_ 00034 and CULC22_00029 to CULC22_00032). The direct repeats of this locus are 29 bp in length and separated by spacers with variable nucleotide sequences that are completely different in both *C. ulcerans *strains. The number of CRISPR spacers is also different in both strains, whereas the *cas *genes and the consensus sequences of the CRISPRs are identical (Table 3).

**Table 3:** Structural features of CRISPR loci detected in the *C. ulcerans *genomes

Name	No. of *cas *genes	No. of spacers	CRISPR size	CRISPR consensus sequence in both genomes
CRISPR809_I	4	28	29 bp	CTTTTCTCCGCGTACGCGGAGGTAGTTCC
CRISPR22_I	4	38	29 bp	

CRISPR809_II	6	12	36 bp	ACCTCAATGAAAGGCTGCGACCGAAGCCGCAGCGAC
CRISPR22_II	6	10	36 bp	

CRISPR809_III	0	67	29 bp	CTTTTCTCCGCGTATGCGGAGGTAGTTCC
CRISPR22_III	0	32	29 bp	

Similar structural features were observed for the second array of CRISPRs in the two *C. ulcerans *genomes (Table 3). CRISPR locus II is flanked by six *cas *genes (CULC809_00109 to CULC809_00114 and CULC22_00106 to CULC22_00111). The consensus sequence of this CRISPR has a length of 36 bp, separated by either 12 spacers in *C. ulcerans *809 or 10 spacers in *C. ulcerans *BR-AD22. The spacers present in *C. ulcerans *809 are different again to those located in the corresponding locus of the *C. ulcerans *BR-AD22 genome. In contrast to CRISPR loci I and II, a third putative CRISPR region in *C. ulcerans *809 and *C. ulcerans *BR-AD22 is not specified by the presence of *cas *genes in the direct proximity. The CRISPR of this genomic region has a length of 29 bp, and the number of spacer sequences revealed the largest variation between both *C. ulcerans *strains, with 67 spacers present in *C. ulcerans *809 and 32 spacers in the genome of *C. ulcerans *BR-AD22 (Table 3). The detection of CRISPRs in the genome of *C. ulcerans *and the sequence variations of the CRISPR loci suggests the use of these molecular genetic markers for a more precise and high-resolution typing of closely related *C. ulcerans *strains from clinical specimens and animal reservoirs. A macroarray-based hybridization method, named spacer oligonucleotide typing ("spoligotyping"), has already been developed to study the polymorphism of spacer sequences in CRISPR loci of distinct ribotypes from epidemic *C. diphtheriae *isolates [38].

### Comparative analysis of the predicted gene content of the *C. ulcerans *genomes

**Figure 4:**
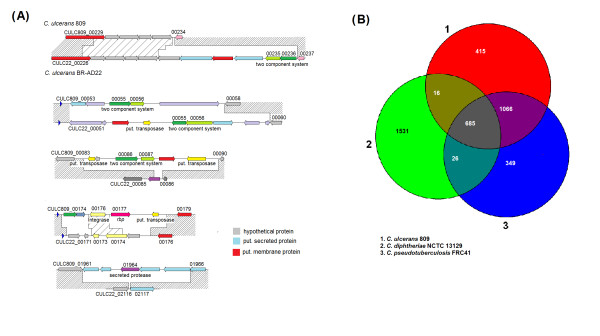
**Intra- and inter-species comparison of the predicted gene content of the *C. ulcerans *genomes**. **(A)**, Selected examples of genomic regions comprising strain-specific genes in *C. ulcerans*. Orthologous gene regions are shaded gray. **(B)**, Venn diagram comparing the gene content of *C. ulcerans *809, *C. diphtheriae *NCTC 13129 and *C. pseudotuberculosis *FRC41. The Venn diagram shows the number of shared and species-specific genes among the three corynebacterial genomes.

The initial analyses of genomic features and the genome architecture of *C. ulcerans *809 and *C. ulcerans *BR-AD22 revealed considerable similarities between the sequenced genomes. The very close relationship of both strains is also evident when calculating the common gene repertoire by reciprocal best BLASTP matches with the EDGAR software [33]. Both *C. ulcerans *strains share a common set of 2,076 genes and are therefore characterized by small numbers of strain-specific genes, named singletons in this study [33]. As most strain-specific genes of the animal isolate *C. ulcerans *BR-AD22 were assigned to the additional prophage-like regions ΦCULC22II¸ ΦCULC22III and ΦCULC22IV, only 92 coding regions of this strain were finally regarded as singletons *sensu stricto*, of which 13 genes were annotated with putative physiological functions (Table 4). This group of singletons includes four genes coding for typical two-component signal transduction systems consisting of a sensor histidine kinase and a DNA-binding response regulator. One two-component system is encoded by the genes CULC22_00235 and CULC22_ 00236 that are located downstream of CULC22_00237 coding for a putative glycerol-3-phosphate transporter (Figure 4A). The genes of the second two-component system (CULC22_00055 and CULC22_00056) are located adjacent to a remnant of a transposase gene (CULC22_00054), suggesting a former event of horizontal gene transfer in this genomic region (Figure 4A). Other singletons of *C. ulcerans *BR-AD22 encode putative enzymes with unknown specificities, such as CULC22_02221 (SGNH-hydrolase family protein) and CULC22_02229 (esterase-lipase family protein). Two genes encoding surface-anchored proteins with LPxTG motif, including the *spaD *gene for the major pilin subunit of an adhesive pilus structure, were also detected as singletons (Table 4). The SpaD protein of *C. ulcerans *BR-AD22 differs in its amino acid sequence when compared with the functional counterpart CULC809_01952 from *C. ulcerans *809, demonstrating that the adhesive pili of the two *C. ulcerans *strains vary significantly in the primary sequence of their major pilins that in principle constitute the shaft of the corynebacterial pilus structure [39].

**Table 4:** Deduced functions of strain-specific genes in *C. ulcerans *809 and *C. ulcerans *BR-AD22

Identifier	Gene	G+C content	Proposed function of deduced protein
CULC809_00055	*nreB*	51.2%	Sensor histidine kinase (two-component system)
CULC809_00056	*nreC*	48.0%	Response regulator (two-component system)
CULC809_00086	*tcsS5*	57.1%	Sensor histidine kinase (two-component system)
CULC809_00087	*tcsR5*	51.1%	Response regulator (two-component system)
CULC809_00176	*intC*	53.1%	Phage-related integrase
CULC809_00177	*rbp*	45.1%	Putative ribosome binding protein
CULC809_01822	-	45.3%	Surface-anchored protein
CULC809_01940	-	52.9%	Surface-anchored protein
CULC809_01952	*spaD*	46.7%	Surface-anchored protein (fimbrial subunit)
CULC809_01964	*vsp2*	49.3%	Venome serine protease

CULC22_00055	*tcsR5*	50.8%	Response regulator (two-component system)
CULC22_00056	*tcsS5*	48.1%	Sensor histidine kinase (two-component system)
CULC22_00171	-	46.8%	DNA-binding transcriptional regulator
CULC22_00173	*intC*	48.7%	Phage-related integrase
CULC22_00174	-	51.1%	Helix-turn-helix domain protein
CULC22_00235	*tcsR1*	50.3%	Response regulator (two-component system)
CULC22_00236	*tcsS1*	52.9%	Sensor histidine kinase (two-component system)
CULC22_01271	*tetR4*	52.7%	TetR-family transcriptional regulator
CULC22_02106	*spaD*	45.1%	Surface-anchored protein (fimbrial subunit)
CULC22_02199	-	48.6%	Surface-anchored protein
CULC22_02221	-	49.0%	SGNH-hydrolase family protein
CULC22_02229	-	61.3%	Esterase-lipase family protein
CULC22_02230	-	62.3%	DNA-binding transcriptional regulator

Furthermore, the search for singletons by reciprocal best BLASTP matches with EDGAR revealed 90 strain-specific genes for the human isolate *C. ulcerans *809, of which 10 were annotated with putative functions (Table 4). The genome annotation revealed two gene pairs coding for two-component systems as singletons, including CULC809_00086 and CULC_00087 that are flanked by transposase genes (Figure 4A). The *vsp2 *gene (CULC809_01964) coding for a secreted serine protease and the *rbp *gene (CULC809_00177) encoding a putative ribosome-binding protein were also recognized as singletons in the genome of *C. ulcerans *809. Both gene products represent candidate virulence factors of *C. ulcerans *809 (see below). Interestingly, the *rbp *gene is located between a gene coding for a putative phage integrase (CULC809_00176) and a transposase gene (CULC809_00178) and is moreover specified by the low G+C content of 45.1% (Table 4), suggesting the horizontal transfer of this gene to *C. ulcerans *809. In summary, the detection and functional assignments of singletons indicates that the repertoire of potential virulence factors of the sequenced *C. ulcerans *strains is different in the two selected isolates from a human and animal source, respectively.

### Inter-species comparison of the gene content detected in the *C. ulcerans *genomes

The gene content of *C. ulcerans *809 was compared in respect to encoded proteins with those of *C. pseudotuberculosis *FRC41 and *C. diphtheriae *NCTC 13129. This comparative content analysis showed that 685 predicted proteins of *C. ulcerans *809 (31.4% of the total number of predicted proteins) share homologs in the genomes of the closely related species (Figure 4B). The number of core genes is remarkably low, as a similar calculation with genomic data from pathogenic and non-pathogenic corynebacteria, including *C. diphtheriae*, *Corynebacterium jeikeium, Corynebacterium efficiens*, and *Corynebacterium glutamicum*, revealed 835 genes as a conserved corynebacterial backbone [40]. These numbers indicate a larger variation in the deduced gene repertoires from *C. ulcerans*, *C. pseudotuberculosis *and *C. diphtheriae *than initially expected when considering their close phylogenetic relationship. Nevertheless, the conserved genetic backbone detected in both comparative studies comprises genes for the basic cellular machineries, such as the components involved in DNA replication, DNA repair, transcription, and protein biosynthesis, the conserved corynebacterial regulatory systems [41], the components of the central carbon and energy metabolism, and of biosynthesis routes for amino acids, cofactors, purines, and pyrimidines, as well as the machinery involved in cell wall formation [40,42].

Interestingly, *C. ulcerans *and *C. diphtheriae *share only 16 genes, which are not present in the genome of *C. pseudotuberculosis *(Figure 4B). According to the genome annotations, most genes of this group encode putative transporters and secreted proteins with unknown functions. On the other hand, *C. ulcerans *and *C. pseudotuberculosis *share 1066 homologous genes (Figure 4B), indicating that these species are more closely related to each other than to *C. diphtheriae*. The physiological functions encoded by this set of genes may also reflect differences in the lifestyle of the animal pathogens and the human pathogen *C. diphtheriae*. The group of homologous coding regions of *C. ulcerans *and *C. pseudotuberculosis *includes 733 genes with unknown function. However, the functional analysis of the remaining genes revealed that they mainly code for transport systems, including a variety of permeases and ABC-type transporters for the uptake of metal ions, indicating an adaptation of both species to the availability of trace elements in their ecological niche. Moreover, genes encoding the subunits of urease and accessory proteins of urease were only detected in the genomes of *C. ulcerans *and *C. pseudotuberculosis*, which is consistent with previous data describing these species as urease-positive, whereas *C. diphtheriae *was tested negative for urease activity [20,43].

As expected, the *pld *gene encoding the sphingomyelin-degrading phospholipase D was found among the subset of genes homologous for *C. ulcerans *and *C. pseudotuberculosis *[18,43]. Phospholipase D represents the major virulence factor of *C. ulcerans *and *C. pseudotuberculosis *and facilitates the persistence and spread of these bacteria within the mammalian hosts [44,45]. The *cpp *gene encoding the "corynebacterial protease CP40" was also detected in the genome of *C. ulcerans*. This secreted protein was described previously as a protective antigen of *C. pseudotuberculosis *[46,47]. As the comparison of the gene content among the three species of the *C. diphtheriae *cluster revealed notable differences in the repertoire of virulence factors, the genome sequences of *C. ulcerans *809 and *C. ulcerans *BR-AD22 were searched for the presence of additional genes probably contributing to the virulence of these strains.

### Detection of genes encoding candidate virulence factors in *C. ulcerans *809 and *C. ulcerans *BR-AD22

To extend the view on proteins probably contributing to the virulence of *C. ulcerans *809 and *C. ulcerans *BR-AD22, the annotated proteomes of both strains were screened for protein precursors with N-terminal secretion signals [48] and proteins containing a C-terminal LPxTG motif allowing their anchoring to the bacterial cell wall [49]. This bioinformatic search revealed twelve candidate virulence factors that are common to both strains and two additional proteins encoded only in the genome of *C. ulcerans *809 (Table 5). In addition to the *tspA *and *vsp1 *gene products representing secreted proteins of the serine protease type in both strains, the *vsp2 *gene (CULC809_01964) encodes an additional extracellular serine protease in *C. ulcerans *809. This enzyme family can show a wide range of pathogenic potentials when interacting with tissue components of the host or with components of the host's defense system [50]. The redundancy of the corresponding enzymatic activities in *C. ulcerans *809 might promote the interaction of the pathogen with the host and the survival of the bacterium in an unfavorable environment.

**Table 5:** Overview of candidate virulence factors detected in the *C. ulcerans *genomes

ID in 809	ID in BR-AD22	Gene	Proposed function of deduced protein	**LP×TG**^**a**^
CULC809_00177	-	*rbp*	Ribosome-binding protein	none
CULC809_01974	CULC22_02125	*cpp*^b^	Corynebacterial protease CP40^b^	none
CULC809_00040	CULC22_00038	*pld*	Phospholipase D	none
CULC809_01949	CULC22_02103	*spaF*	Surface-anchored protein (pilus subunit)	LPKTG
CULC809_01950	CULC22_02104	*spaE*	Surface-anchored protein (pilus subunit)	LPLTG
CULC809_01952	CULC22_02106	*spaD*	Surface-anchored protein (pilus subunit)	LPMTG
CULC809_01979	CULC22_02130	*spaC*	Surface-anchored protein (pilus subunit)	LPLTG
CULC809_01980	CULC22_02131	*spaB*	Surface-anchored protein (pilus subunit)	LARTG
CULC809_01133	CULC22_01148	*rpfI*	Rpf interacting protein	none
CULC809_01521	CULC22_01537	*cwlH*	Cell wall-associated hydrolase	none
CULC809_00434	CULC22_00437	*nanH*	Sialidase precursor (neuraminidase H)	none
CULC809_00509	CULC22_00515	*vsp1*	Venome serine protease	none
CULC809_01964	-	*vsp2*	Venome serine protease	none
CULC809_01848	CULC22_02007	*tspA*	Trypsin-like serine protease	none

^a ^Predicted LPxTG motif used for anchoring of the protein to the cell wall

^b ^Data presented in this manuscript indicates a function as endoglycosidase. The gene should therefore be renamed as *ndoE*.

Another potential virulence factor of *C. ulcerans *is the extracellular neuraminidase NanH (Table 5). Some enzymatic properties of this enzyme were characterized in a previous study indicating that this thermo-labile protein has a temperature optimum of 37°C and hydrolyses substrates such as horse serum glycoproteins [51]. The homologous enzyme from *C. diphtheriae *was characterized previously and shown to contain neuraminidase and *trans*-sialidase activities [52,53]. In principle, neuraminidases are a distinct class of glycosyl hydrolases that catalyze the removal of terminal sialic acids from various glycoconjugates and contribute to the recognition of sialic acids exposed on host cell surfaces, whereas *trans*-sialidases can be used for the decoration of various acceptor molecules on the cell surface to enable the invasion of host cells under certain conditions [54]. Therefore, microbial neuraminidases and *trans*-sialidases have the general capacity to modify the ability of host cells to respond to bacterial infections and are thus of importance for any pathogenic microorganism.

Furthermore, two gene clusters detected in each *C. ulcerans *genome are considered to encode adhesive pilus structures that are covalently anchored to the corynebacterial cell wall and probably mediate the initial adhesion of the pathogen to host tissues (Table 5). Different types of adhesive pili can presumably allow the pathogen to interact with different receptors on the host cell surface and to facilitate the delivery of virulence factors and intracellular invasion. The genetic organization of the first gene cluster is similar to the *spaDEF *gene region of *C. diphtheriae *NCTC 13129 encoding the SpaDEF pilus [55]. This adhesive pilus of *C. diphtheriae *is composed of the major pilin SpaD, the minor pilin subunit SpaE and the tip protein SpaF. The assembly of the SpaDEF precursors into a high-molecular-weight pilus structure requires the pilus-specific sortases SrtB and SrtC that are encoded within the *spaDEF *gene region of *C. diphtheriae *[55]. Likewise, the sortase genes *srtB *and *srtC *are present in the *spaDEF *regions of *C. ulcerans *809 and *C. ulcerans *BR-AD22 and probably involved (in conjunction with the housekeeping sortase gene *srtD *[56]) in the assembly of a distinct pilus structure on the cell surface of these strains.

The second pilus gene cluster detected in the genomes of the *C. ulcerans *strains consists of the *spaBC *genes and the single sortase gene *srtA*. The *spaB *gene encodes a minor pilus protein, whereas the *spaC *gene codes for a tip protein. The respective adhesive pilus of *C. ulcerans *thus lacks a major pilin subunit, unless it can be replaced by the major pilin SpaD of the SpaDEF pilus. However, comprehensive studies in *C. diphtheriae *demonstrated that the adhesive pili of strain NCTC 13129 are independently assembled and are morphologically distinct [55,57,58], suggesting that a replacement of the major pilin subunit is unlikely in *C. ulcerans*. Interestingly, the adherence of *C. diphtheriae *to human pharyngeal epithelial cells can be mediated also by the minor pilin SpaB of the SpaABC pilus [59]. Therefore, it is likely that homodimeric or heterodimeric SpaB/SpaC proteins are anchored covalently to the cell surface of *C. ulcerans *809 and *C. ulcerans *BR-AD22 and provide tight contact between the bacterial cells and the host tissue in the absence of a pilus shaft [60].

Moreover, genes encoding homologs of the resuscitation-promoting factor interacting protein DIP1281 (RpfI/RipA) and the cell wall-associated hydrolase DIP1621 (CwlH) from *C. diphtheriae *NCTC 13129 were detected in the genomes of *C. ulcerans *809 and *C. ulcerans *BR-AD22 (Table 5). Both conserved enzymes are involved in organizing the corynebacterial cell surface and contribute-probably indirectly-to the adhesion of *C. diphtheriae *to epithelial cells and the subsequent internalization of this pathogen [61-63].

### Prominent virulence factors of *C. ulcerans*: phospholipase D, endoglycosidase EndoE and the Shiga toxin-like ribosome-binding protein Rbp

**Figure 5:**
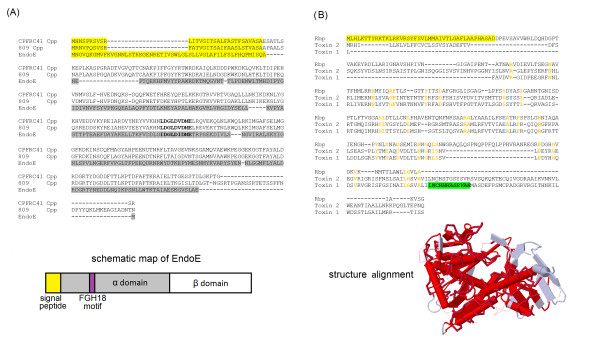
**Sequence analyses of prominent virulence factors detected in the genome of *C. ulcerans *809**. **(A)**, Analysis of the corynebacterial protease CP40 (Cpp). An amino acid sequence alignment of the corynebacterial proteases CP40 from *C. pseudotuberculosis *FRC41 and *C. ulcerans *809 with the α domain of endoglycosidase EndoE from *E. faecalis *is shown. Predicted signal peptides are colored in yellow; predicted protein segments belonging to the α domain of EndoE are shaded gray. The catalytic FGH18 motif of EndoE is indicated by bold letters. The domain organization of EndoE is shown schematically below the sequence alignment. **(B)**, Analysis of the corynebacterial ribosome-binding protein (Rbp). An amino acid sequence alignment of Rbp from *C. ulcerans *809 with A chains of the Shiga-like toxins SLT-1 and SLT-2 from *E. coli *is shown. Conserved amino acids are highlighted in orange, while the conserved catalytic residues are highlighted in blue. The predicted signal peptide of Rbp is labeled yellow; the retranslocation domain of SLT-1 is marked as a green box. The similarity between Rbp and the A chain of SLT-1 is also shown as a 3-D model presented below the sequence alignment. Structural similarities between both proteins are indicated in red.

A well-established virulence factor of *C. ulcerans *is the toxic phospholipase D (Pld) that shows amino acid sequence similarity to secreted phospholipases from *C. pseudotuberculosis *and *Arcanobacterium haemolyticum *[18]. While sequence homologs of the Pld enzyme are not found elsewhere in bacterial species, a similar enzyme is produced as an exotoxin in *Loxosceles *spiders. The spider and bacterial enzyme were shown to hydrolyze albumin-bound lysophosphatidylcholine, yielding the lipid mediator lysophosphatidic acid, a known inducer of pro-inflammatory responses [64]. The *pld *gene was detected in both *C. ulcerans *genomes, which confirms previous PCR assays performed during the initial characterization of the two isolates [15,25]. Moreover, the *cpp *gene (CULC809_01974 and CULC22_02125) encoding "corynebacterial protease CP40" is present in the genome sequences of *C. ulcerans *809 and *C. ulcerans *BR-AD22 (Table 5). The homologous enzyme from *C. pseudotuberculosis *was identified as a protective antigen against caseous lymphadenitis [46,47] and shown to be of the serine protease type as the purified protein revealed proteolytic activity in a gelatine SDS-PAGE assay [65]. On the other hand, database searches did not identify any active-site homologies with other serine proteases and protease activity was undetectable in culture supernatants of *C. pseudotuberculosis *[65]. As presented in Figure 5A, the amino acid sequences of Cpp from *C. ulcerans *809 and *C. pseudotuberculosis *FRC41 revealed striking similarities to the α-domain of the extracellular endoglycosidase EndoE from *Enterococcus faecalis *[66]. The three proteins contain the conserved FGH18 motif assigning them to the glycosyl hydrolases of family 18 that includes enzymes with endo-β-*N*-acetylglucosaminidase activity [67]. EndoE from *E. faecalis *is a two-domain protein that is characterized by two distinct activities involved in the degradation of *N*-linked glycans from ribonuclease B and the hydrolysis of the conserved glycans on IgG [66]. The latter activity of the enzyme was assigned exclusively to the β-domain of the EndoE protein, suggesting that the homologous counterparts from *C. ulcerans *have only a single activity as endoglycosidase. Nevertheless, both *C. ulcerans *strains are probably able to interact with the mammalian host by glycolytic modulation of host glycoproteins.

As described above, the group of singletons detected in the genome sequence of *C. ulcerans *809 comprises the *rbp *gene (CULC809_00177) that encodes a putative ribosome-binding protein containing the Pfam domain 00161 named "ribosome inactivating protein" [68]. The deduced amino acid sequence of *rbp *shows weak similarity to the A chains of the Shiga-like toxins SLT-1 and SLT-2 from *Escherichia coli *(Figure 5B). The SLT-1 protein of *E. coli *belongs to the ribosome-binding protein type II family that is characterized by low similarity on the amino acid sequence level and, on the other hand, by a highly conserved tertiary structure of the family members [69]. The ribosome-binding protein identified in the *C. ulcerans *809 genome shares only 24% identity with the A chain of SLT-1, but comprises all highly conserved amino acid residues needed for the catalytic *N*-glycosidase activity (Figure 5B). Moreover, an *in silico *comparison of the tertiary structures of Rbp and the A chain of SLT-1 demonstrates significant structural similarities of both proteins (Figure 5B). SLT-1 is usually composed of a catalytic A chain that is non-covalently associated with a pentamer of B chains [70]. The B subunits of SLT-1 are essential for binding a specific glycolipid receptor and the subsequent translocation of the toxin into the endoplasmatic reticulum (ER) of the host cell. The ER-targeting sequence of SLT-1 leads to retranslocation of the catalytic domain into the cytosol and the subsequent inhibition of protein biosynthesis by depurination of a single adenosine residue in the 28S rRNA of the eukaryotic ribosome [70]. *In vitro*, SLT-1 has been demonstrated to induce apoptosis in endothelial cells isolated from various anatomical sites [71]. The analysis of the amino acid sequence of Rbp revealed the lack of the ER-targeting sequence at the C-terminal end of the protein from *C. ulcerans *809. However, as *C. ulcerans *can probably persist as a facultative intracellular pathogen in mammalian host cells, a retranslocation of the toxin into the cytosol is not necessary. The secretion of the putative toxin into the cytosol of the host cell is supported instead by a typical signal sequence at the N-terminus of the protein (Figure 5B). As the enzymatic activity of the ribosome-binding protein Rbp leads to inhibition of protein biosynthesis, as is the case with diphtheria toxin [72], *C. ulcerans *809 could have mimicked the systemic symptoms of diphtheria in the infected patient without carrying a lysogenic β-corynephage [54].

## Conclusions

The analysis of the complete genome sequences from *C. ulcerans *809 and *C. ulcerans *BR-AD22 provides detailed insights into the genome architecture and the repertoire of candidate genes contributing to the virulence of these strains. Both *C. ulcerans *isolates differ in the number of prophage-like elements in the genome and lack sequences with similarity to the β-corynephage encoding the diphtheria toxin. Therefore, the sequenced *C. ulcerans *isolates can be regarded in principle as "non-toxigenic" with respect to the synthesis of diphtheria toxin. This result of the genome sequence annotation is remarkable, as previous PCR studies and toxigenicity assays with *C. ulcerans *809 suggested the presence of a diphtheria-like toxin in this strain that was isolated from a patient with pulmonary infection [15]. However, the comparison of *tox *genes showed differences in the amino acid sequences of the diphtheria toxin from *C. diphtheriae *and *C. ulcerans *isolates from extrapharyngeal infections [2,3]. This data indicated that the diphtheria toxin sequences from *C. ulcerans *isolates are less well conserved than their counterparts from *C. diphtheriae *[3]. Moreover, the examination of diagnostic PCR assays for the detection of the *tox *gene in clinical samples revealed atypical results in some *C. ulcerans *strains [73], indicating that a reliable diagnostic is necessary for the precise characterization of *C. ulcerans *strains from clinical specimens [74]. The absence of the diphtheria toxin gene in *C. ulcerans *809 moreover explains why the medical treatment of the elderly patient with a diphtheria antitoxin was unsuccessful [15].

The integration site of the β-corynephage is located at a tRNA^Arg ^gene in the genomes of *C. diphtheriae *and *C. ulcerans *[75,76]. A remnant of a β-like phage was detected at this genomic position in the genome sequence of *C. ulcerans *809, and the *rbp *gene encoding a ribosome-binding protein is present in the immediate vicinity. The low G+C content of the *rbp *gene is suggestive of the horizontal transfer of this coding region to *C. ulcerans *809. Whether the *rbp *gene was a former part of a corynephage or associated with an adjacent transposable element cannot be deduced from the current data and remains to be elucidated. The Rbp protein of *C. ulcerans *809 revealed structural similarity to the A chain of the Shiga-like toxin SLT-1 from *E. coli *[69]. The SLT-1 protein has been implicated in the pathogenesis of acute renal failure [71] that was also diagnosed in the elderly Brazilian woman with pulmonary infection [15]. Therefore, the Rbp protein of *C. ulcerans *809 may represent a prominent virulence factor by inhibiting the protein biosynthesis in host cells due to the putative ribosome-binding activity.

Interestingly, the *rbp *gene is absent in the genome sequence of *C. ulcerans *BR-AD22 that was isolated from an asymptomatic dog [25]. As the current knowledge of *C. ulcerans *is biased due to the predominant recovery of toxigenic strains from respiratory diphtheria-like illnesses in humans, future work should include strains from extrapharyngeal specimens and particularly from various animal sources. The sequencing of more *C. ulcerans *genomes from different habitats will open the way to the pan-genomic level of comparative genomics. A comparative approach with a larger set of sequenced *C. ulcerans *genomes may help to gain insights into the distinctive features of strains from human and animal sources and to describe the proposed zoonotic transmission of this pathogen in more detail.

It is moreover necessary to clarify the physiological role of the predicted "corynebacterial protease CP40". In a previous study, the homologous enzyme from *C. pseudotuberculosis *was assigned to the serine protease family due to its associated proteolytic activity although no protease activity could be detected in *C. pseudotuberculosis *culture supernatants [65]. In contrast to this data, the *in silico *analysis of the domain organization of CP40 in this study and previous phylogenetic data of glycosyl hydrolases strongly indicate that this protein is an endoglycosidase with similarity to the α domain of EndoE from *E. faecalis *[66]. The α domain of EndoE hydrolyzes the glycans on RNase B, which could be important for the pathogenesis and persistence of a bacterium during human infections [66]. We therefore suggest renaming the corresponding genes from *C. ulcerans *and *C. pseudotuberculosis *as "*ndoE*". In summary, the comparative analysis of two complete *C. ulcerans *genomes provides new valuable information on known virulence factors and detected novel candidate genes probably contributing to the virulence of this species. According to the genome annotation, the repertoire of prominent virulence factors from *C. ulcerans *809 comprises the phospholipase D, the neuraminidase NanH, the novel ribosome-binding protein Rbp, and the endoglycosidase EndoE (formerly named corynebacterial protease CP40).

## Methods

### Bacterial strains and growth conditions

*C. ulcerans *809 was originally isolated in Rio de Janeiro from a bronchoalveolar lavage sample of an elderly woman with a fatal pulmonary infection and a history of leg skin ulcers [15]. *C. ulcerans *BR-AD22 was previously obtained from a nasal sample of an asymptomatic female dog kept in an animal shelter in the metropolitan area of Rio de Janeiro [25]. Both isolates were assigned to the species *C. ulcerans *by taxonomic assays and sequencing of the 16S rDNA. The clinical history of the patient and the examination of the respective dog were described in detail in previous studies [15,25]. Both *C. ulcerans *strains were routinely grown at 37°C in brain-heart-infusion (BHI) broth or on Columbia agar supplemented with 5% sheep blood.

### Preparation of chromosomal DNA for genome sequencing

The preparation of chromosomal DNA from *C. ulcerans *809 and *C. ulcerans *BR-AD22 was performed as described previously [21]. Briefly, 50-ml aliquots of bacterial cultures grown for 48-72 h in BHI broth were centrifuged at 4°C and 2,000 × *g *for 20 min. The resulting cell pellets were resuspended in 0.6 ml Tris/NaCl buffer [10 mM Tris (pH 7.0), 10 mM EDTA, 300 mM NaCl] and transferred to VK01 Precellys lysing tubes. The bacterial cells were lysed by means of a Precellys 24-Dual Tissue Homogenizer using two cycles of 6,500 rpm for 15 sec with an interval of 30 sec. The chromosomal DNA was subsequently purified by extraction with phenol/chloroform/isoamyl alcohol (25:24:1) and precipitated with ethanol. DNA concentrations were determined with a Tecan Infinite 200 Microplate Reader.

### Genome sequencing of *C. ulcerans *809 and *C. ulcerans *BR-AD22

Single-stranded template DNA libraries for genome sequencing of *C. ulcerans *809 und *C. ulcerans *BR-AD22 were established by using 5 μg of purified chromosomal DNA for each strain. The preparation of the DNA libraries was carried out according to standard protocols from Roche Applied Science. The DNA concentration of the resulting libraries was measured with the Agilent RNA 6000 Nano Kit. DNA sequencing was performed with the Genome Sequencer FLX Instrument and Titanium chemistry (Roche Applied Science). The genomic sequences were assembled with the Newbler Assembler software (version 2.3) and the results were documented in the respective 454 Newbler Metrics files. The automatic assembly of genomic sequences from *C. ulcerans *809 yielded 14 large (> 500 bases) and 8 small contigs, together composed of 250,786 assembled reads representing 106,993,163 sequenced bases. The assembly of genomic sequences from *C. ulcerans *BR-AD22 led to 25 large and 3 small contigs, based on 178,615 assembled reads representing the total number of 59,757,327 sequenced bases. The subsequent gap closure process was facilitated by *in silico *predictions of the contig order that were computed by the related reference contig arrangement tool r2cat using the *C. pseudotuberculosis *FRC41 genome sequence as a reference and the default parameters of the integrated *q*-gram filter [21,26]. All matching regions were displayed in an interactive synteny plot and oriented automatically according to their matches, using a sliding window approach that determines the position of a contig on the reference genome [26]. The remaining gaps in the genome sequences were closed by PCR assays with Phusion hot start high-fidelity DNA polymerase (Finnzymes) and genomic template DNAs. The PCR assays were performed according to standard protocols from Finnzymes using 1 M betain for efficient denaturation of DNA secondary structures. The amplified DNA fragments linking individual contigs were sequenced by IIT Biotech (Bielefeld, Germany). All contigs and additional DNA sequences were uploaded into the Consed program [77] to finish the genome sequences of *C. ulcerans *809 and *C. ulcerans *BR-AD22.

### Annotation and bioinformatic analysis of the genome sequences

The annotations of the assembled genome sequences of *C. ulcerans *809 and *C. ulcerans *BR-AD22 were performed with the GenDB system that supports automatic annotation strategies and manual data curation [27]. In the GenDB annotation system, a combined gene prediction strategy was executed by means of REGANOR, GLIMMER 2.1 and the CRITICA program suite along with postprocessing by the RBSfinder tool. The predicted proteins of *C. ulcerans *were functionally characterized by automated searches in public databases, including SWISS-PROT, TrEMBL, Pfam, TIGRFAM, KEGG, COG, CDD and Interpro. Finally, an automated functional annotation was performed using default parameters. The origin of chromosomal DNA replication was predicted with the Ori-Finder program [28]; CRISPRs were detected with the CRISPRFinder tool [36]. Both tools were applied with their recent web versions using default parameters. Metabolic properties of the sequenced *C. ulcerans *strains were deduced from *in silico *reconstructions of metabolic networks with the software CARMEN using metabolic pathway information from the KEGG database and from manually curated SBML templates [78]. The predicted *C. ulcerans *proteins were mapped onto the SBML templates by means of bidirectional best BLASTP hits using the scoring matrix BLOSUM62 and the user-defined E-value cutoff of 1 × 10^-10^. The synteny between corynebacterial genomes was calculated by the EDGAR software with default parameters [33]. Comparative analysis of the predicted gene content of *C. ulcerans *809 and *C. ulcerans *BR-AD22 was also performed with the EDGAR platform. This analysis was based on the calculation of BLAST score ratio values using the pre-calculated corynebacterial master cutoff of 77 [33]. Secreted proteins were detected with SignalP 3.0 using the default settings for gram-positive bacteria [48]. Multiple amino acid sequence alignments were generated with the Clustal W 1.82 program [79]. The comparison of tertiary structures of proteins was computed by means of the Dali server using default parameters. This server computes structural alignments between two protein structures using the DaliLite-pairwise option (version 3.1) [80]. The PDB file for Rbp was calculated with the SWISS-Model workspace automated modeling mode using the SLT-1 A1 chain (PDB code 1dm0) as reference input [81]. The genome sequences of *C. ulcerans *809 (CP002790) and *C. ulcerans *BR-AD22 (CP002791) have been deposited in the GenBank database.

## Authors' contributions

ET sequenced and annotated the *C. ulcerans *genomes and prepared the manuscript. AA and PP participated in genome annotation with GenDB. JS implemented the GenDB project and provided bioinformatic support. PV participated in the gap closure process. SS, SA and FD purified the genomic DNAs. The comparative genome analysis was supported by AB, AM, VA, MS and AS. CS, LS, PS, AD, RH and AM isolated and characterized the *C. ulcerans *strains prior to this study and participated in the comparative analysis. AT coordinated and supervised the project and the comparative analysis. All authors read and approved the final version of the manuscript.
